# Inter- and intra-rater reliability of video-documented Pirani Böhm Sinclair score: A potential method to screen for signs of recurrence in children with idiopathic clubfoot?

**DOI:** 10.1177/18632521251349437

**Published:** 2025-06-24

**Authors:** Åsa Thelaus, Salik Kashif, Eva Broström, Alaric Aroojis, Steven Frick, Stephanie Böhm, Josefine E Naili

**Affiliations:** 1 Department of Women’s and Children’s Health, Karolinska Institutet, Stockholm, Sweden; 2 Pediatric Orthopedic Department, Karolinska University Hospital, Stockholm, Sweden; 3 Department of Orthopedic and Spine Surgery, Khyber Girls Medical College, Pershawar, Pakistan; 4 MTI Hayatabad Medical Complex Phase 4, Khyber Girls Medical College, Phase 5 Hayatabad, Peshawar, Pakistan; 5 Motion Analysis Lab, Karolinska University Hospital, Stockholm, Sweden; 6 Lilavati Hospital, PD Hinduja Hospital, Bai Jerbai Wadia Hospital for Children, Mumbai, India; 7 Atrium Health Wake Forest University School of Medicine, Charlotte, NC, USA

**Keywords:** Congenital talipes equinovarus, foot deformity, clubfoot, PBS-score, relapse

## Abstract

**Purpose:**

The recurrence rate in children with idiopathic clubfoot is high. The Pirani Böhm Sinclair score is a clinical tool screening for signs of recurrence. This study examined the inter- and intra-rater reliability of video-documented Pirani Böhm Sinclair score.

**Methods:**

In two pediatric orthopedic centers, children with idiopathic clubfoot aged 4–15 years were consecutively included and filmed using a standardized protocol to complete the Pirani Böhm Sinclair score. Four pediatric orthopedic surgeons with extensive experience treating clubfoot patients viewed the videos and scored the feet according to the Pirani Böhm Sinclair score. Intra-class correlation coefficient and Kappa statistics were used to determine reliability.

**Results:**

Fifty-five children with 85 clubfeet were included (54% bilateral). A subset of 30 videos was reassessed at a separate occasion by two of the four raters. Fleiss unweighted Kappa showed substantial agreement between all raters for the item “early heel rise,” and moderate agreement for all remaining items except “swing phase supination” which was fair. Intra-class correlation coefficient for the total Pirani Böhm Sinclair score was almost perfect for both agreement and consistency on a group level and for each rater pair, respectively. Examination of intra-rater reliability showed substantial to almost perfect agreement for five items for both raters, but not the same five items.

**Conclusions:**

This study highlights the potential use of video-documented Pirani Böhm Sinclair score. A high level of agreement was found for the item “early heel rise” where reduced playback speed and repeated viewing may aid assessment, while a low level of agreement was observed for the clinically important item “walking supination.” Further development of the scoring instructions is required.

## Introduction

Clubfoot is the most common congenital foot deformity with an estimated incidence of 1–2 cases/1000 births.^1,2^ The clubfoot deformity consists of a combination of cavus, adductus, varus, and equinus.^3^ The current gold standard treatment, the Ponseti method, consists of manipulation following a defined protocol and serial casting, often accompanied by tenotomy of the Achilles tendon.^4,5^ Despite successful initial correction, 11%–48% of the patients experience a recurrence of deformity during childhood and adolescence.^6^ A recurrent clubfoot does not resolve spontaneously but requires treatment to prevent worsening. Treatment of recurrent foot deformity may consist of recasting, bracing, soft tissue procedures, such as the Tibialis anterior tendon transfer, or bony surgery, including osteotomies or arthrodesis.^7–9^ All forms of recurrence impair the child in daily activities to various degrees.^10–12^

There are several scores and outcome measures to assess clubfoot deformity. The Pirani score and the Dimeglio score are primarily used for the initial classification of the severity of deformity in the infant and non-ambulatory child.^3,13^ These have been reported to be able to predict prognosis for long-term outcomes and risk of recurrence.^14^ The Pirani Böhm Sinclair (PBS) score was developed for the ambulatory child to grade the severity of recurrence. High inter-rater reliability has been reported when scoring was carried out in a live setting.^15^ The PBS score consists of seven items assessing and observing the child in sitting, standing, and walking, thus both in static and dynamic.^15^ Clubfoot Assessment Protocol score is another clinical tool including assessment of passive mobility, muscle strength, morphology, and motion quality.^16^ The score is validated for children up to 7 years of age. The PAVER score (Plantarflexion, Adduction, Varus, Equinus, Rotation) has been used for classification of the late-presenting untreated clubfoot and for predicting ease of reduction.^17^ In 2021, a Core Outcome Set, including the PBS score, for the study of clubfoot was published.^18^

According to the literature, the goal for successful club foot treatment is more than a plantigrade and pain-free foot. Several studies and consensus statements define a fully corrected foot as having a covered talar head, neutral heel, and dorsiflexion that allows normal gait.^18–20^ The course of treatment for children with clubfoot, following the initial treatment, includes repeated clinical visits throughout childhood and adolescence to screen for signs of recurrence. The clinical follow-up programs are dependent on the accessibility to care and geographical location, and may be time-consuming for both health care and families. As such, frequent follow-ups are associated with high costs for the family because of time away from work.^21^

The PBS score includes both dynamic and static evaluation, but requires no advanced equipment. Video documentation can be performed with a smartphone or a tablet, making the material available for assessment remotely and over time. The feasibility of scoring signs of clubfoot recurrence based on video-documented material has not been evaluated in previous research. Nor has the reliability of the PBS-score, among orthopedic surgeons with little or no experience in using the score, been examined.

This study aimed to examine the inter- and intra-rater reliability of video-documented PBS-score, both with regards to individual items and the total score. It was hypothesized that individual items requiring manual examination would display lower level of agreement as compared to items without manual examination.

## Methods

### Study design and study participants

This cross-sectional observational cohort study was conducted in accordance with the Helsinki declaration, and approved by the Swedish ethical review authority Dnr 2022-03647-01 and the ethical review board of the Hayatabad Medical Complex, Pakistan HMC-QAD-F-00 approval number 1210. All participating children and caregivers provided informed consent. Children with idiopathic clubfoot were recruited from two pediatric orthopedic centers: Astrid Lindgrens Children’s Hospital, Karolinska University Hospital in Stockholm, Sweden, and Hayatabad Medical Complex, Orthopaedic Unit, Peshawar, Pakistan. A total sample of 55 children aged 4–15 years were consecutively included during a routine visit to the outpatient clinic between January and December 2023 (Table 1). Inclusion criteria included age 4–15 years, a diagnosis of idiopathic clubfoot, the ability to walk unassisted for 10 m, and the ability to communicate in Swedish (for the Swedish cohort), Urdu or Pashto (for the Pakistani cohort). Exclusion criteria included secondary clubfeet due to syndromes or neuromuscular conditions. The methodology and findings of this study have been reported in line with the STROBE (Strengthening the Reporting of Observational Studies in Epidemiology) checklist.^22^

**Table 1.** Background characteristics of included children with clubfoot, and the four independent raters.

Study cohort	Sweden	Pakistan	All participants
Participants, *n* (%)	41 (75)	14 (25)	55
Years of age, median (range)	7 (4–15)	Not available	Not available
Girls, *n* (%)	9 (22)	5 (36)	14
Bilateral clubfoot, no. of feet (%)	44 (70)	16 (73)	60
Unilateral clubfoot, no. of feet (%)	19 (30)	6 (27)	25
Clubfoot videos assessed (*n*)	63	22	85
Participating raters (*n* = 4)			
Female, *n* (%)	2 (50)		
Years of age, median (range)	53 (41–59)		
Years of practice^a^, median (range)	22 (7–26)		
Country of residence			
Sweden (*n*)	2		
USA (*n*)	1		
India (*n*)	1		

*n*: number.

a Years of practice as a pediatric orthopedic surgeon.

### Sample size estimation

Based on pilot data, an a priori sample size calculation was performed. A total sample of 80 club feet would reach sufficient statistical power (>0.8) to determine the level of intra-class correlation (ICC) when testing the null hypothesis that the ICC is 0 with a two-way random-effects model.

### The PBS score

The PBS score is a validated clinical score developed to evaluate clubfoot deformity in walking children.^15^ It consists of a total of seven dynamic and static items scoring the foot of a child in standing, walking, and sitting. There are five dichotomous items and two 4-scale step items. The sum score ranges from 7 to 18, where 7 corresponds to a foot without any deformity and 18 is a foot with fixed deformity. For definitions and graphic illustrations of the included items, we refer the reader to the original publication.^15^

### Standardized video-documentation protocol

A protocol for standardized video documentation of the PBS score was developed by a subset of the authors (JEN, SB, ÅT; Supplement 1). This protocol included both general instructions about the videos and item-specific instructions. The videos should be recorded with a neutral background using a high-quality camera held in a horizontal position. Each item included instructions about the position of the camera in relation to the foot. Videos of items in standing (1 and 2) were to be recorded for a minimum of 10 s, and walking items had a requirement of at least 10 consecutive steps; walking supination, hereafter termed “swing phase supination” (item 3), was filmed using a frontal view, “early heel rise” (item 4) was filmed using a sagittal view. Active and passive ankle dorsiflexion (items 5 and 6) was filmed using a sagittal view (displaying the lateral malleolus) with the knee flexed to 90°. For the passive ankle dorsiflexion, a rigid plate was placed under the foot. “Subtalar abduction” (item 7) was filmed using a frontal/transversal view, including all of the lower leg and foot for reference (Supplement 1). All videos were sorted to follow the order of the items in the PBS score (1–7), and merged into one file for each clubfoot.

### Rating protocol

A rating protocol was developed by a subset of the authors (JEN, SB, ÅT; Supplement 2). The raters were asked to score the feet according to this protocol. Accompanying the boxes to score each item was also a box to check if the item was deemed “unable to assess” (i.e., due to poor quality of the video or other reasons for not being able to score that item). Following the rating according to the PBS-score, the raters were asked to provide a treatment recommendation (data not reported in the present study).

### Participating raters

An international group of four pediatric orthopedic surgeons (AA, SB, SF, and ÅT) with 22 (7–26) years of experience in treating clubfoot was recruited (Table 1). Prior to the rating session, raters were instructed to familiarize themselves with the rating protocol and the original article of the PBS-score.^15^ This was carried out to reduce the risk of measurement bias.

### The inter-rater reliability rating sessions

**Figure 1.**
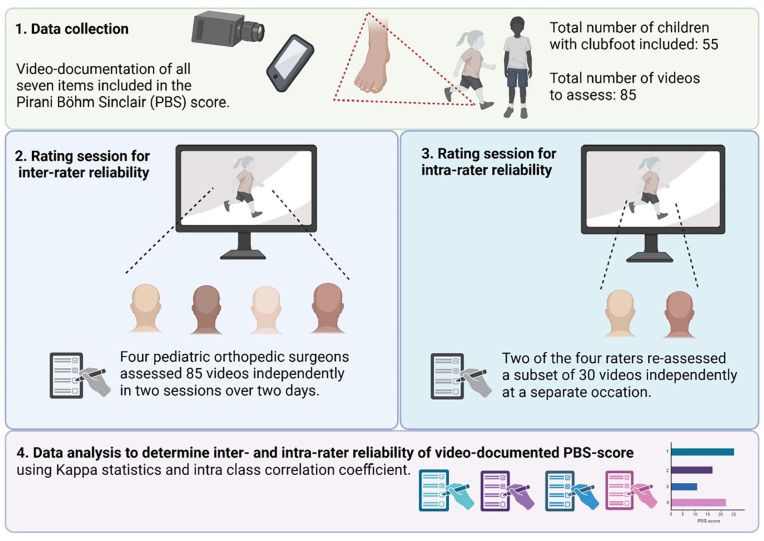
Flow chart of data collection, rating session procedures, and data analysis to evaluate inter- and intra-rater reliability of video-documented Pirani Böhm Sinclair score. Figure created with biorender.com.

Over 2 days (4 h/day), live rating sessions were carried out in May 2024 (Figure 1). Each set of videos was viewed over a course of 6 min; each item was reviewed twice, the first time at normal playback speed, and the second time at reduced playback speed (0.67×). After viewing all seven items, the raters were given 2 min to complete the rating protocol, including their treatment recommendation. During the rating sessions, no discussion among raters was allowed. A few example videos were presented to all raters before the actual rating session started. The example videos provided the raters with real experience of the order of the videos, the length, speed, and also the time provided to fill out the scoring sheet.

### The intra-rater reliability rating session

Four weeks after the first rating session was conducted, a separate rating session was carried out. The session was conducted using a similar room and set-up (Figure 1). A subset of videos was re-assessed by two of the four raters (1 and 4). During the rating session, no discussion among raters was allowed.

### Statistical analysis

Statistical analysis was performed using the R version 4.4.1 (R Core Team, Vienna, Austria), the Psych package, and the Statistical Package for Social Sciences, version 26 (SPSS, Inc., Chicago, IL, USA) with *p* < 0.05 determining statistical significance. Demographics and study participant characteristics were described using median, minimum, maximum, frequency, and/or percent. To evaluate whether there was a difference in median total PBS score between the two cohorts, the Mann–Whitney *U* test was used. Fleiss’ unweighted Kappa was calculated to evaluate agreement between all four raters.^23^ Unweighted Kappa statistics were calculated for all items and all rater pairs. Weighted Kappa statistics were calculated using quadratic weighting for the items with ordinal variables: “passive ankle dorsiflexion” and “subtalar abduction.” Compared to the linearly weighted Kappa statistic (Supplement 3), the quadratically weighted Kappa statistic results in higher values when disagreements are small.^24,25^ ICC was calculated for consistency and agreement for the total PBS-score and each item for each pair of raters, and between all four raters as a group. Level of agreement using Kappa statistics was interpreted according to Landis and Koch: >0.00–0.20 slight; 0.21–0.40 fair; 0.41–0.60 moderate; 0.61–0.80 substantial; and 0.81–1.00 almost perfect level of agreement.^26^ Kappa statistics and ICC are presented as means and 95% confidence intervals.

## Results

In total, 85 videos, each representing one clubfoot, were reviewed by all four raters (Table 2). A subset of 30 videos was reassessed at a separate occasion by two of the four raters.

**Table 2.** The median and range of total PBS score for each of the four raters, presented for the Swedish and Pakistani cohorts, respectively, and for the total cohort.

Rater	Total PBS score of video-assessed clubfeet		
	Sweden (*n* = 63)	Pakistan (*n* = 22)	All videos (*n* = 85)
	Median (range)		
Rater 1	10 (7–15)	14 (9–18)	11 (7–18)
Rater 2	9 (7–16)	17 (10–18)	11 (7–18)
Rater 3	9 (7–14)	15 (10–17)	10 (7–17)
Rater 4	8 (7–13)	15 (9–18)	9 (7–18)
	Median (range)		Difference between cohorts *p*
Total PBS-score	9 (7–16)	15 (9–18)	<0.001

*n*: number; PBS: Pirani Böhm Sinclair.

### Missing data

Of the 85 videos of clubfeet, two videos were excluded due to not containing the correct material. In 12 videos, it was not possible to calculate the total PBS score, due to missing video material of individual items and items rated “unable to assess” (active dorsiflexion: *n* = 1, subtalar abduction: *n* = 2, and early heel rise: *n* = 3). However, the data for the existing items for these videos were included in the individual item analysis. A total of three individual items were not scored by the raters, where the checkbox was left blank without any reason provided.

### Inter-rater reliability

Fleiss unweighted Kappa showed substantial agreement between all four raters for the item “early heel rise,” and moderate agreement for all remaining items except for “swing phase supination,” which was fair (Table 3). Unweighted Cohen’s Kappa for dichotomous items varied between rater pairs (Table 3). Kappa values were higher between raters 1 and 2, and raters 3 and 4, respectively (Table 3). Weighted quadratic Cohen’s Kappa displayed substantial to almost perfect agreement in the item “passive ankle dorsiflexion” containing four scale steps (Table 3). Two rater pairs (pair 2 and 3, and pair 3 and 4) showed almost perfect agreement in the item “subtalar abduction.” ICC for the total PBS score was almost perfect for both agreement and consistency on a group level, and for each rater pair, respectively.

**Table 3.** Inter-rater reliability of the video-documented PBS score was performed among four independent raters assessing a total of 85 videos. Inter-rater reliability was evaluated using Fleiss’ unweighted Kappa, unweighted Cohen’s Kappa, weighted Cohen’s Kappa, and intra-class correlation values for all items of the PBS-score. Values are presented for all raters together and for rater pairs separately.

Item	Fleiss’ unweighted Kappa (upper and lower limits of 95% CI)	Unweighted Cohen’s Kappa (upper and lower limits of 95% CI)					
	All raters	Rater (1 versus 2)	Rater (1 versus 3)	Rater (1 versus 4)	Rater (2 versus 3)	Rater (2 versus 4)	Rater (3 versus 4)
Heel varus	0.60 (0.51–0.69)	0.59 (0.43–0.75)	0.77 (0.61–0.92)	0.65 (0.47–0.83)	0.54 (0.38–0.70)	0.54 (0.38–0.70)	0.58 (0.38–0.78)
Standing supination	0.48 (0.39–0.57)	0.59 (0.41–0.76)	0.52 (0.32–0.72)	0.47 (0.26–0.68)	0.30 (0.13–0.47)	0.35 (0.18–0.52)	0.80 (0.62–0.99)
Swing phase supination	0.38 (0.29–0.46)	0.60 (0.43–0.78)	0.28 (0.15–0.41)	0.34 (0.18–0.50)	0.32 (0.17–0.47)	0.35 (0.18–0.53)	0.57 (0.38–0.77)
Early heel rise	0.69 (0.60–0.78)	0.77 (0.63–0.91)	0.67 (0.51–0.83)	0.62 (0.46–0.79)	0.69 (0.54–0.85)	0.66 (0.50–0.82)	0.72 (0.57–0.88)
Active ankle dorsiflexion	0.52 (0.43–0.61)	0.57 (0.40–0.73)	0.65 (0.48–0.81)	0.36 (0.22–0.50)	0.61 (0.44–0.78)	0.61 (0.45–0.77)	0.41 (0.25–0.57)
Passive ankle dorsiflexion	0.45 (0.39–0.50)	0.54 (0.41–0.67)	0.45 (0.31–0.60)	0.42 (0.28–0.56)	0.52 (0.39–0.66)	0.36 (0.23–0.49)	0.39 (0.24–0.53)
Subtalar abduction	0.41 (0.35–0.47)	0.18 (0.06–0.30)	0.25 (0.09–0.40)	0.35 (0.17–0.54)	0.61 (0.47–0.76)	0.41 (0.27–0.56)	0.56 (0.40–0.71)
		Weighted (quadratic) Cohen’s Kappa (upper and lower limits of 95% CI)					
		Rater (1 versus 2)	Rater (1 versus 3)	Rater (1 versus 4)	Rater (2 versus 3)	Rater (2 versus 4)	Rater (3 versus 4)
Passive ankle dorsiflexion		0.82 (0.74–0.91)	0.75 (0.65–0.86)	0.76 (0.67–0.86)	0.76 (0.64–0.88)	0.74 (0.64–0.84)	0.74 (0.64–0.84)
Subtalar abduction		0.45 (0.24–0.65)	0.56 (0.34–0.79)	0.72 (0.51–0.92)	0.84 (0.76–0.93)	0.71 (0.55–0.86)	0.85 (0.76–0.95)
		Intra-class correlation for total PBS score (upper and lower limits of 95% CI)					
	Agreement all raters	Rater (1 versus 2)	Rater (1 versus 3)	Rater (1 versus 4)	Rater (2 versus 3)	Rater (2 versus 4)	Rater (3 versus 4)
Agreement	0.82 (0.72–0.89)	0.86 (0.76–0.91)	0.85 (0.77–0.90)	0.82 (0.55–0.91)	0.81 (0.67–0.89)	0.78 (0.26–0.91)	0.85 (0.66–0.92)
Consistency	0.86 (0.81–0.90)	0.87 (0.81–0.92)	0.85 (0.77–0.90)	0.87 (0.80–0.92)	0.84 (0.75–0.89)	0.87 (0.80–0.92)	0.88 (0.81–0.92)

CI: confidence interval; PBS score: Pirani Böhm Sinclair score.

Unweighted Cohen’s Kappa was used to determine inter-rater reliability for dichotomous items. Weighted Kappa was used to determine inter-rater reliability for items with four scale steps. Kappa values were interpreted according to (REF): 0–0.20 poor, 0.21–0.40 fair, 0.41–0.60 moderate, 0.61–0.80 substantial, and 0.81–1.00 almost perfect inter-rater reliability.

### Intra-rater reliability

A subset of 30 videos from the total cohort was randomly selected to be reassessed. In this subset, there were no missing data, and no items were deemed “unable to assess” by the two raters (raters 1 and 4).

For rater 1, Fleiss unweighted Kappa showed substantial to almost perfect agreement for five items, moderate agreement for the item “heel varus,” and fair agreement for the item “standing supination” (Table 4). For rater 4, Fleiss unweighted Kappa showed substantial agreement for five items, almost perfect agreement for the item “swing phase supination,” and moderate agreement for the item “subtalar abduction” (Table 4). Weighted quadratic Cohen’s Kappa showed almost perfect agreement for both the four-scale items for rater 1, and substantial to almost perfect agreement for rater 4 (Table 4). ICC for both raters was almost perfect concerning agreement and consistency.

**Table 4.** Intra-rater reliability was evaluated for two raters (raters 1 and 4) using unweighted Cohen’s Kappa, weighted Cohen’s Kappa, and intra-class correlation values for all items of the PBS score. Video assessment to evaluate intra-rater reliability was performed on a subset of 30 videos from the total cohort of 85 videos.

Item	Unweighted Cohen’s Kappa (upper and lower limits of 95% CI)	
	Rater 1	Rater 4
Heel varus	0.60 (0.31–0.89)	0.66 (0.36–0.96)
Standing supination	0.33 (0.00–0.66)	0.61 (0.21–1.00)
Swing phase supination	1.00 (1.00–1.00)	0.85 (0.65–1.00)
Early heel rise	0.71 (0.46–0.97)	0.73 (0.50–0.96)
Active ankle dorsiflexion	0.71 (0.45–0.97)	0.79 (0.58–1.00)
Passive ankle dorsiflexion	0.64 (0.44–0.84)	0.67 (0.47–0.87)
Subtalar abduction	0.74 (0.38–1.00)	0.50 (0.14–0.85)
	Weighted (quadratic) Cohen’s Kappa (upper and lower limits of 95% CI)	
	Rater 1	Rater 4
Passive ankle dorsiflexion	0.91 (0.85–0.97)	0.88 (0.76–0.99)
Subtalar abduction	0.97 (0.91–1.00)	0.74 (0.42–1.00)
	Intra-class correlation for total PBS-score (upper and lower limits of 95% CI)	
	Rater 1	Rater 4
Agreement	0.93 (0.86–0.97)	0.95 (0.89–0.97)
Consistency	0.93 (0.87–0.97)	0.95 (0.89–0.98)

CI: confidence interval; PBS-score: Pirani Böhm Sinclair score.

Unweighted Cohen’s Kappa was used to determine intra-rater reliability for dichotomous items. Weighted Kappa was used to determine intra-rater reliability for items with four scale steps. Kappa values were interpreted according to (REF): 0–0.20 poor, 0.21–0.40 fair, 0.41–0.60 moderate, 0.61–0.80 substantial, and 0.81–1.00 almost perfect intra-rater reliability.

## Discussion

This study examined the inter- and intra-rater reliability of video-documented PBS score. To this end, four pediatric orthopedic surgeons scored a total of 85 videos of clubfeet according to the PBS score. Results of the individual item analysis demonstrate that the item “early heel rise” can reliably be assessed using video documentation, while the item “swing phase supination” cannot. Contrary to the hypothesis, the two items involving manual examination (“passive dorsiflexion” and “subtalar abduction”) did not display poorer agreement than items without manual examination. These items are scored using a four-point scale with scale steps corresponding to 5° of range of motion. Two rater pairs showed almost perfect agreement for the item “subtalar abduction.” For the item “passive dorsiflexion,” one rater pair demonstrated almost perfect agreement. Results of the total PBS score showed that ICC was almost perfect for both agreement and consistency on a group level, and for each rater pair, respectively.

It is worth pointing out that throughout the analysis, the highest rater pair agreement was not observed between the two raters working in the same department. The ICC values of the total PBS score reported in the present study were similar to those of the original study, where assessments according to the PBS score were performed in a live setting. The total PBS score has been proposed to be able to guide treatment decisions, although no clear cutoff values have been reported.^15^ Results of this study showed greater diversity on item-specific levels compared to the original study. This finding is of particular clinical importance since the individual items “swing phase supination” and “early heel rise” in combination with other clinical outcome measures often are determinants of treatment decisions for the recurrent clubfoot.^8^ This is also relevant as equinus is reportedly one of the first signs of recurrence.^8,9^

“Swing phase supination” is a clinical feature that is often decisive for considering anterior tibial tendon transfer surgery.^6–8,27^ The inter-rater reliability of this item was only fair among the four raters. When this item was reassessed at a separate occasion, intra-rater reliability Kappa statistics showed an almost perfect level of agreement for both raters individually. In the original article of the PBS score, the instruction to score “swing phase supination” is to have the child walk toward the examiner.^15^ If the forefoot supinates at the time of active dorsiflexion in the swing phase, then the item is scored as present. However, if a child presents with a fixed deformity (i.e., is scored as having a “standing supination”), it is not clear whether that disqualifies the child from being able to be assessed as having also the dynamic “swing phase supination.” This ambiguity may explain the observed lower level of agreement for this item across rater pairs and for all four raters on a group level, as compared to the high level of intra-rater reliability. A clear definition of what constitutes a swing phase supination is needed.

Weighted Kappa statistics were calculated using quadratic weighting for the items with ordinal variables. Compared to the linearly weighted Kappa statistics, the quadratically weighted Kappa results in higher values when disagreements are small.^24,25,28^ Results of the present study show that disagreements were small, which is highlighted by the higher quadratically weighted Kappa values, both for the inter-rater reliability and the intra-rater reliability. This raises questions whether the 5° scale steps in the items “passive dorsiflexion” and “subtalar abduction” need to be revised to 10° scale steps if used for video-documented and possibly also live scoring. In the original study of the PBS score, unweighted kappa values for these items ranged from 0.60 to 1.00 (“passive dorsiflexion”) and 0.26 to 0.77 (“subtalar abduction”), respectively.

Today, routine clinical visits in different areas of health care have been replaced by video meetings and home-based assessments carried out by the caregivers.^29,30^ Video assessments could potentially be a viable alternative to reduce, but not altogether replace, the number of in-person clinical appointments to screen for signs of clubfoot recurrence and facilitate assessments of patients in remote locations or countries with limited access to pediatric orthopedic surgeons. This method could potentially aid in the early detection of recurrence and thereby prevent extensive treatment. However, further development of the scoring instructions is warranted. Future studies should also seek to determine clear cutoff values of the PBS-score, or combinations of items present that require attention or specific treatment.

The strengths of this study include the examination of children from two different countries and an adequate sample size. The included children displayed a wide range of severity of deformity (PBS-score 7–18), which reduces the risk for prevalence issues.^24^ The wide range of present deformities also increases the generalizability of findings. Furthermore, the four raters represent three different countries, and as such, different practices and traditions concerning treatment and care for children with clubfoot. There was a varying degree of experience in using the PBS score, which can be viewed both as a strength and a limitation. One of the raters has been involved in developing the score and co-authored the original publication, which may entail a unique “insider” perspective and pre-understanding of item testing during the development of the score. This rater and the department colleague rater have been using the score in daily clinical practice since 2017, and reporting it to the national registry for clubfoot at specific time points.^31^ The remaining two raters were novices to the score and used it for the first time during the rating session. They were provided with written instructions and the original publication ahead of the rating session. All raters participated in the viewing of the example videos ahead of the actual rating session. Interestingly, the two raters more experienced in using the PBS score did not demonstrate a higher level of agreement in the pair-wise Kappa analysis, as compared to any other combination of raters. This implies that scoring may be influenced by factors beyond the time spent using the score. The specific factors remain speculative. However, it can be assumed that the environment in which a physician trains and works establishes a gold standard for managing clubfoot patients, thereby shaping their understanding of the most essential clinical findings.

## Conclusion

Findings of this study highlight the potential use of video-documented PBS score. Results showed a high level of agreement for the total PBS score, and for the item “early heel rise” where reduced playback speed and repeated viewing may aid assessment. A low level of agreement was observed for the clinically important item “swing phase supination.” Following further development of the scoring instructions, this method may offer a reduction of repeated in-person clinical visits and a potential alternative for patients with limited access to pediatric orthopedic surgeons.

## Supplemental Material

sj-docx-2-cho-10.1177_18632521251349437 – Supplemental material for Inter- and intra-rater reliability of video-documented Pirani Böhm Sinclair score: A potential method to screen for signs of recurrence in children with idiopathic clubfoot? Supplemental material, sj-docx-2-cho-10.1177_18632521251349437 for Inter- and intra-rater reliability of video-documented Pirani Böhm Sinclair score: A potential method to screen for signs of recurrence in children with idiopathic clubfoot? by Åsa Thelaus, Salik Kashif, Eva Broström, Alaric Aroojis, Steven Frick, Stephanie Böhm and Josefine E Naili in Journal of Children’s Orthopaedics

sj-docx-3-cho-10.1177_18632521251349437 – Supplemental material for Inter- and intra-rater reliability of video-documented Pirani Böhm Sinclair score: A potential method to screen for signs of recurrence in children with idiopathic clubfoot? Supplemental material, sj-docx-3-cho-10.1177_18632521251349437 for Inter- and intra-rater reliability of video-documented Pirani Böhm Sinclair score: A potential method to screen for signs of recurrence in children with idiopathic clubfoot? by Åsa Thelaus, Salik Kashif, Eva Broström, Alaric Aroojis, Steven Frick, Stephanie Böhm and Josefine E Naili in Journal of Children’s Orthopaedics

sj-docx-4-cho-10.1177_18632521251349437 – Supplemental material for Inter- and intra-rater reliability of video-documented Pirani Böhm Sinclair score: A potential method to screen for signs of recurrence in children with idiopathic clubfoot? Supplemental material, sj-docx-4-cho-10.1177_18632521251349437 for Inter- and intra-rater reliability of video-documented Pirani Böhm Sinclair score: A potential method to screen for signs of recurrence in children with idiopathic clubfoot? by Åsa Thelaus, Salik Kashif, Eva Broström, Alaric Aroojis, Steven Frick, Stephanie Böhm and Josefine E Naili in Journal of Children’s Orthopaedics

sj-pdf-1-cho-10.1177_18632521251349437 – Supplemental material for Inter- and intra-rater reliability of video-documented Pirani Böhm Sinclair score: A potential method to screen for signs of recurrence in children with idiopathic clubfoot? Supplemental material, sj-pdf-1-cho-10.1177_18632521251349437 for Inter- and intra-rater reliability of video-documented Pirani Böhm Sinclair score: A potential method to screen for signs of recurrence in children with idiopathic clubfoot? by Åsa Thelaus, Salik Kashif, Eva Broström, Alaric Aroojis, Steven Frick, Stephanie Böhm and Josefine E Naili in Journal of Children’s Orthopaedics
