# Supplementary material for: Inter- and intra-rater reliability of video-documented Pirani Böhm Sinclair score: A potential method to screen for signs of recurrence in children with idiopathic clubfoot?
Source: J Child Orthop. 2025 Jun 24;19(4):312–20. doi: 10.1177/18632521251349437 (PMC12187707; doi:10.1177/18632521251349437)
Supplement: sj-docx-2-cho-10.1177_18632521251349437 – Supplemental material for Inter- and intra-rater reliability of video-documented Pirani Böhm Sinclair score: A potential method to screen for signs of recurrence in children with idiopathic clubfoot? [file sj-docx-2-cho-10.1177_18632521251349437.docx]

**Supplement 1**

**Standardized video analysis protocol**

- A **neutral background** is desirable for all video recordings.
- **Camera with good quality**: Mobile phone (**held in a horizontal position**) or video camera, steady recordings are of great importance (i.e., use of stand/tripod if possible).

**Item 1 – Standing: Hind foot varus**

The child is standing still, **facing away from the camera**. Camera viewpoint should include the knees, calf, and feet. Film for at least 10 seconds.

**Item 2 – Standing: Supination**

The child is standing still, **facing towards the camera**. Camera viewpoint should include the knees, calf, and feet. Film for at least 10 seconds.

**Item 3 – Walking: Swing phase supination**

The child is filmed with a **stationary frontal view** while walking bare foot **for at least 10 consecutive steps**. Camera viewpoint should be equivalent to an examiner kneeling or sitting on a low chair.

**Item 4 – Walking: Early heel rise**

The child is filmed with a **stationary side** **view** while walking bare-foot for at least 10 consecutive steps (NB! all 10 steps will not all fit into the camera viewpoint, but allows for “normal gait pattern”). Camera viewpoint should be equivalent to an examiner kneeling or sitting on a low chair.

**Item 5 – Sitting: Active ankle dorsiflexion**

The child is sitting on a firm surface, raised to allow for the lower legs and ankle to hang freely. The knee joints should be in 90 degrees’ flexion. The child performs his/her maximum active dorsiflexion (angle between the fibula and foot-sole). Film the entire sequence of range of motion with a **stationary side** **view**. Camera viewpoint should include all of the lower leg and foot for reference.

**Item 6 – Sitting: Passive ankle dorsiflexion**

The child is sitting on a firm surface, raised to allow for the lower legs and ankle to hang freely. The knee joints should be in 90 degrees’ flexion. The examiner uses a plate to passively evaluate the range of dorsiflexion (angle between the fibula and foot-sole). Film the entire sequence of range of motion with a **stationary side** **view**. Camera viewpoint should include all of the lower leg and foot for reference.

**Item 7 – Sitting: Subtalar abduction**

The child is sitting on a firm surface, raised to allow for the lower legs and ankle to hang freely. The knee joints should be in 90 degrees’ flexion. The examiner uses his/her thumb to fixate the talar head, abducts the foot, to evaluate the range of subtalar abduction (angle between long axis of tibia and long axis of the first MT). Film the entire sequence of range of motion with a **stationary** **frontal/transversal view and from above**. Camera viewpoint should include all of the lower leg and foot for reference.
