# Supplementary material for: Inter- and intra-rater reliability of video-documented Pirani Böhm Sinclair score: A potential method to screen for signs of recurrence in children with idiopathic clubfoot?
Source: J Child Orthop. 2025 Jun 24;19(4):312–20. doi: 10.1177/18632521251349437 (PMC12187707; doi:10.1177/18632521251349437)
Supplement: sj-docx-3-cho-10.1177_18632521251349437 – Supplemental material for Inter- and intra-rater reliability of video-documented Pirani Böhm Sinclair score: A potential method to screen for signs of recurrence in children with idiopathic clubfoot? [file sj-docx-3-cho-10.1177_18632521251349437.docx]

**Supplement 2**

Patient ID _____________________________________

Examiner name ________________________________

Circle your option

|  | | |  | **Video assessment not possible to perform** | |
| --- | --- | --- | --- | --- | --- |
| **Item** | **Left** | **Right** |  | **Left** | **Right** |
| Hindfoot varus | 1 = absent  2 = present | 1 = absent  2 = present |  | □ | □ |
| Standing forefoot supination | 1 = absent  2 = present | 1 = absent  2 = present |  | □ | □ |
| Dynamic forefoot supination | 1 = absent  2 = present | 1 = absent  2 = present |  | □ | □ |
| Early heel rise | 1 = absent  2 = present | 1 = absent  2 = present |  | □ | □ |
| Active ankle dorsiflexion | 1 = >0  2 = <0 | 1 = >0  2 = <0 |  | □ | □ |
| Passive ankle dorsiflexion | 1 = >10  2 = 5-10  3 = 0-5  4 = <0 | 1 = >10  2 = 5-10  3 = 0-5  4 = <0 |  | □ | □ |
| Passive subtalar abduction | 1 = >10  2 = 5-10  3 = 0-5  4 = <0 | 1 = >10  2 = 5-10  3 = 0-5  4 = <0 |  | □ | □ |
| **Total PBS Score  (7-18)** |  |  |  |  |  |

Patient ID _____________________________________

Examiner name ________________________________

**What would you recommend for this patient?**

Circle any that apply.

| **Left foot** | Do nothing/ observe | Cast | Bracing | Lengthen Achilles tendon | Anterior Tendon Transfer | Other soft tissue procedure | Bony procedure |
| --- | --- | --- | --- | --- | --- | --- | --- |

Other: ________________________________________________________________

| **Right foot** | Do nothing/ observe | Cast | Bracing | Lengthen Achilles tendon | Anterior Tendon Transfer | Other soft tissue procedure | Bony procedure |
| --- | --- | --- | --- | --- | --- | --- | --- |

Other: ________________________________________________________________

Additional comments/remarks: _____________________________________________________________________

_____________________________________________________________________

_____________________________________________________________________
