# Supplementary material for: Inter- and intra-rater reliability of video-documented Pirani Böhm Sinclair score: A potential method to screen for signs of recurrence in children with idiopathic clubfoot?
Source: J Child Orthop. 2025 Jun 24;19(4):312–20. doi: 10.1177/18632521251349437 (PMC12187707; doi:10.1177/18632521251349437)
Supplement: sj-docx-4-cho-10.1177_18632521251349437 – Supplemental material for Inter- and intra-rater reliability of video-documented Pirani Böhm Sinclair score: A potential method to screen for signs of recurrence in children with idiopathic clubfoot? [file sj-docx-4-cho-10.1177_18632521251349437.docx]

| **Supplement 3.** Linear weighted Cohen’s Kappa used to determine inter-rater reliability for items with four scale steps.  Kappa and Intra Class Correlation values were interpreted according to (Landis and Koch, Biometrics 33:159-174, 1977)  0-0.20 poor, 0.21-40 fair, 0.41-0.60 moderate, 0.61-0.80 substantial, and 0.81-1 almost perfect. | | | | | | |
| --- | --- | --- | --- | --- | --- | --- |
|  | **Weighted (linear) Cohen's Kappa**  (Upper and lower limits of 95% CI) | | | | | |
|  | **Examiner  1 vs. 2** | **Examiner  1 vs. 3** | **Examiner  1 vs. 4** | **Examiner  2 vs. 3** | **Examiner  2 vs. 4** | **Examiner  3 vs. 4** |
| **Passive ankle dorsiflexion** | 0.71  (0.60,0.81) | 0.62  (0.50, 0.74) | 0.61  (0.50, 0.73) | 0.66  (0.54, 0.78) | 0.58  (0.46, 0.69) | 0.59  (0.47, 0.70) |
| **Subtalar abduction** | 0.33  (0.15, 0.50) | 0.41  (0.21, 0.61) | 0.55  (0.32, 0.78) | 0.74  (0.64, 0.85) | 0.58  (0.43, 0.74) | 0.73  (0.60, 0.86) |
|  |  | | | | | |
